# Axial compression behavior and design of perforated high-strength steel square hollow section stub columns

**DOI:** 10.1038/s41598-026-53799-4

**Published:** 2026-05-18

**Authors:** Joel T. C. Vanlalnunzira, Lalzuimuani Khiangte, Ricky Lalthazuala

**Affiliations:** Department of Civil Engineering, National Institute of Technology, Mizoram, India

**Keywords:** Compression, Square hollow section, Cold formed steel, high-Strength steel, Perforation, Direct Strength method, Finite element modelling, Engineering, Materials science, Mathematics and computing

## Abstract

Perforations are frequently introduced in steel structural members to accommodate service ducts and reduce structural weight; however, their presence can significantly affect the load-carrying capacity of compression members. This study presents a numerical investigation on the performance of cold-formed high-strength steel (CFHSS) square hollow section (SHS) stub columns having double-sided perforations. Finite element (FE) models were developed and validated against available experimental results for non-perforated high-strength steel (HSS) SHS stub columns and perforated carbon steel SHS stub columns, due to the absence of experimental data for perforated HSS members. This limitation is acknowledged and discussed. A comprehensive set of experimental and numerical column capacities from previous studies and the present parametric analysis were used to evaluate the accuracy of existing codified and proposed design equations, including the Direct Strength Method (DSM). The comparisons show that most current design equations for perforated stub columns provide overly conservative and highly scattered predictions of column capacity for perforated stub columns. To further investigate these effects, a parametric study covering the influence of thickness, perforation shape, size, and height on column capacity was conducted. Among all parameters, the size effect was found to have a significant impact on the stub column capacity. Hence, a modified DSM design equation has been proposed for various perforation size ratios (i.e. diameter of perforation to the width of the column specimen) considering the previous test results and current FE column capacities. A tri-linear equation for the size ratios *d/D ≤ 0.5* and *0.5 < d/D < 0.7* were separately proposed. The proposed design equation demonstrates improved predictive accuracy and reliability within the investigated parameter range and may serve as a useful basis for future development of design provisions for perforated high-strength steel tubular stub columns, subject to further experimental validation.

**Supplementary Information:**

The online version contains supplementary material available at 10.1038/s41598-026-53799-4.

## Introduction

High-strength tubular steel members are increasingly used in modern structural systems such as buildings, bridges, transmission towers, and offshore platforms due to their high strength-to-weight ratio and efficient structural performance. In recent years, the application of high-strength steel (HSS), particularly grades such as S690 and S700, has gained significant attention in structural engineering because it allows for reduced member sizes while maintaining high load-carrying capacity. The use of HSS also improves structural efficiency and material utilization in compression members^1^. Among various steel cross-sections, square hollow sections (SHS) are widely adopted in structural applications due to their high torsional rigidity, uniform stress distribution, and superior aesthetic appearance^2–4^. SHS members are commonly used as columns, truss members, and compression elements in building frames and transmission towers, where efficient structural performance and architectural considerations are required^5^.

In practical structural applications, openings or perforations are often introduced in steel members to facilitate the passage of service ducts, electrical cables, ventilation systems, and mechanical installations^6,7^. Perforations may also be used to reduce structural weight and improve functional integration within buildings. However, the introduction of openings creates local discontinuities in the structural system, leading to stress concentration, stiffness degradation, and changes in load distribution^8–13^. These effects become particularly critical in thin-walled steel members subjected to axial compression, where local buckling often governs structural failure^14,15^. Therefore, understanding the influence of perforation geometry, size, and location on the structural behavior of steel members has become an important topic in structural engineering research.

Fundamental insights into the effects of perforations originate from classical plate studies, where Ritchie and Rhodes^14^ demonstrated that the presence of holes in compressed plates substantially reduces buckling resistance and post-buckling strength, primarily due to stress concentration and stiffness degradation. Early investigations into the behavior of perforated structural components mainly focused on plates and shell structures subjected to compression. One of the earliest contributions was reported by Jullien and Limam^16^, who investigated the buckling behavior of cylindrical shells with openings under axial compression and observed that openings significantly reduce the buckling strength due to local stress redistribution and stiffness reduction. In a related study, Marshall and Nurick^17^ examined progressive buckling behavior in thin-walled square tubes and demonstrated that structural imperfections and geometric discontinuities can accelerate the onset of local buckling and influence the post-buckling response of thin-walled members. Larsson^15^ further investigated orthotropic plates with circular openings and showed that perforation geometry governs the onset of local buckling and failure patterns. Building on these findings, Shanmugam et al.^18^ proposed design formulations for axially compressed perforated plates, establishing a foundation for predicting strength reduction due to openings.

Building on these plate studies, later research focused on the behavior of hollow structural sections and tubular columns with perforations. Ma et al. and Chan^19^ reported that strain hardening in cold-formed high-strength steel stub columns can partially compensate for strength losses caused by imperfections and sectional discontinuities. Research on perforated square hollow and similar hollow stub columns by Dhanalakshmi and Shanmugam^20,21^ showed that opening size and location significantly influence local buckling behavior and axial capacity. Yao et al.^22^ revealed that inelastic local buckling in perforated plates and sections under compression is strongly dependent on perforation geometry, while Umbarkar et al.^8^ confirmed that even a single circular perforation can markedly reduce the axial load-carrying capacity of hollow stub columns. More recent experimental and numerical investigations on perforated cold-formed steel SHS stub columns by several researchers^3,4,6,7,9–11,23–26^ consistently demonstrated that perforations alter stress distribution, trigger premature local buckling, and reduce axial strength, with the severity governed by opening shape, size, and position.

Although extensive research has been conducted on perforated thin-walled members with low yield strength, limited studies have specifically addressed high-strength steel tubular stub columns with perforations. Therefore, a significant research gap exists in the context of high-strength steel (HSS) stub columns with perforations, particularly regarding their axial compression performance, failure mechanisms, and post-buckling response. While numerous studies have focused on cold-formed steel (CFS), aluminum, and stainless-steel members with various perforation geometries, only limited research has addressed the unique challenges posed by HSS—such as S700 grade—under similar conditions. There remains a lack of comprehensive numerical and experimental studies focusing specifically on the influence of perforation geometry, size, spacing, and location in high-strength steel stub columns.

The main novelty of this study lies in the comprehensive investigation of perforated CFHSS SHS stub columns of S700 grade, which has not been adequately addressed in existing literature. The key contributions include: (i) systematic evaluation of perforation shape, size, and height effects under axial compression, (ii) assessment of existing DSM-based design methods including AISI S-100 16^27^, along with modified Direct Strength Method proposed by various researchers^24,28–31^ in this field, in order to assess their applicability and limitations for perforated CFHSS SHS stub column (iii) development of modified DSM-based design equations incorporating perforation effects, and (iv) reliability analysis of the proposed design approach. This combined framework provides new insight into the behavior and design of perforated CFHSS SHS stub columns.

## Numerical modelling

### General

The numerical investigation to be presented in the following sections is based on the complementary experimental investigation by Ma et al.^1^ and Singh & Singh^6^ following non-linear static analysis by various researchers^4,8,13,21,32–38^. The measured geometric dimensions of stub columns reported by Ma et al.^1^ has been utilized to develop FE models as provided in Tables 1 and 2 representing the measured dimensions of perforated stub column reported by Singh & Singh^6^. After successful validation of the FE models with the tested results, a series of parametric studies was conducted to study the effect of perforations on CFHSS SHS stub column.

### Material property

**Fig. 1:**
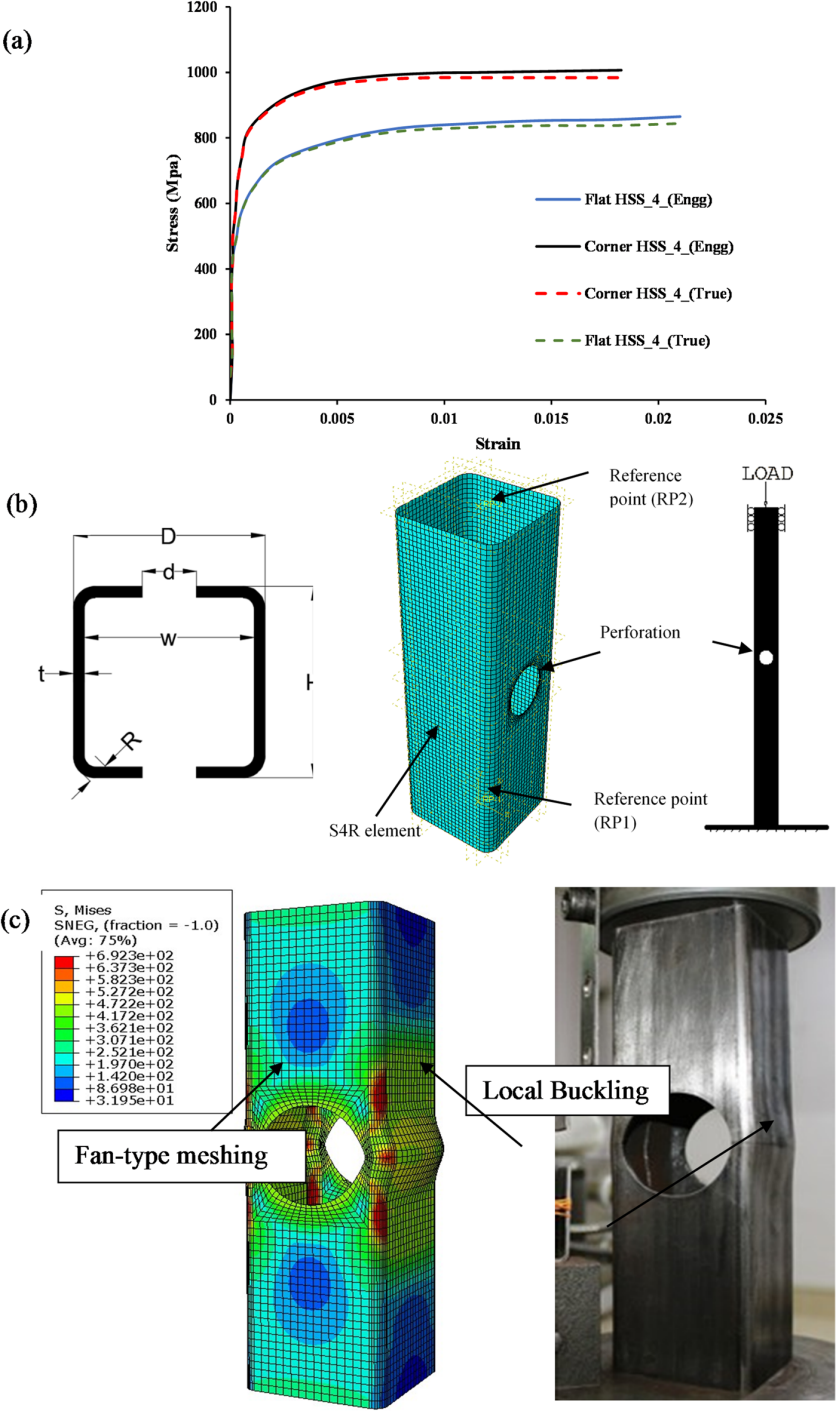
(**a**) Calculated high strength steel S700 stress-strain curve (**b**) FE-mesh with circular perforation with boundary condition and schematic drawing of loading diagram and (**c**) comparison of experimental test by Singh and Singh^6^ and FE failure mode.

The measured engineering stress-strain curves generated from the tensile coupon tests for both flat and corner regions detailed in Ma et al.^1^ have been employed in the numerical models. Figure 1a shows the stress-strain curve of the adopted S700 high strength material property. The key material parameters are provided in Table 3 for flat and corner coupons respectively, in which *E*,* σ*_*0.2*_, *σ*_*u*_, *ε*_*f*_ and n are the Young’s modulus, 0.2% proof stress, ultimate strength, percentage elongation at fracture and strain hardening exponent respectively. The non-linear stress-strain curves have been converted into true stress *σ*_*true*_ and true logarithmic plastic strain *ε*^*pl*^_*true*_, as defined by the Eqs. (1) and (2) respectively, have been further employed in the FE models^11^.

1 $$\:{\sigma\:}_{true}={\sigma\:}_{nom}\left(1+{\epsilon\:}_{nom}\right)$$

2 $$\:{{\epsilon\:}^{pl}}_{true}={ln}\left(1+{\epsilon\:}_{nom}\right)-\frac{{\sigma\:}_{true}}{{E}_{0}}\:\:\:\:\:\:\:\:\:\:\:\:\:\:\:\:\:\:\:\:\:\:\:\:\:\:\:\:\:\:\:\:\:\:\:\:\:\:\:\:\:\:\:\:\:\:\:\:\:\:\:\:\:\:\:\:\:\:\:\:\:\:\:\:\:\:\:\:\:\:\:\:$$

where *σ*_*nom*_ = engineering stress and *ε*_*nom*_ = engineering strain. Poisson’s ratio υ of 0.3 has been considered.

As reported by several researchers [7,11,23,31,39] based on the micro-hardness test, the spread of corner strength enhancement due to cold-forming was found to be concentrated within the vicinity of corner region. Hence, the flat and corner material properties are employed in the flat and corner regions, separately. To account for the spread of cold-forming effects, the corner properties were extended into the adjacent flat regions over a distance of 2*t* from the corner tangent point. This approach is supported by experimental findings reported by J.-L. Ma et al. [1], which show that strain hardening extends beyond the curved corner into the flat elements. The transition between flat and corner material zones was modelled as abrupt for simplicity and in line with common finite element modelling practices.

**Table 1:** Summary of cross-section dimensions of square hollow section from Ma et al.^1^ adopted for validation against finite element FEM Model.

Specimen D x H x t (mm)	Length (L) (mm)	Width(D) (mm)	Thickness (t) (mm)	Depth (H) (mm)	Outer radius (*R*) (mm)	Area (A) (mm^2^)
H120 × 120 × 4	360	120.9	3.92	120.7	8	1792
H160 × 160 × 4	480	160.5	3.97	160.3	10.5	2421
H140 × 140 × 4	420	141.1	3.96	141.1	13	2174.71
V140 × 140 × 6	420	141.1	5.96	140.9	13	3119

**Table 2:** Summary of cross-section dimensions of perforated square hollow section from Singh & Singh^6^ adopted for validation against finite element FEM Model.

	Length(L) (mm)	Width(D) (mm)	Thickness(t) (mm)	Depth(H) (mm)	Inner radius (*r*) (mm)	d/w (mm)
50 × 50 × 2.9d/w0.5 = 1	200.2	49.56	2.89	49.82	2.9	0.5
60 × 60 × 2.6d/w0.5 = 1	199.94	60.04	2.6	60.18	2.6	0.5
60 × 60 × 2.6d/w0.7 = 1	199.94	60.0	2.6	60.18	2.6	0.7

**Table 3:** Basic material property of steel property of HSS S700 obtained from Ma et al.^1^.

Portion	E (GPa)	σ_0.2_ (MPa)	σ_u_ (MPa)	ε_f_ (%)	*N*
Flat	212	719	840	4.3	5.1
Corner	212	897	983	1.6	7.5

### Local geometric imperfection

The initial geometric imperfection was introduced using the first local buckling mode obtained from eigenvalue analysis utilizing the Lanczos Eigen solver from the ABAQUS [40] library. A consistent imperfection amplitude based on Eq. (3) was adopted for all parametric models. The amplitude varies with section geometry through the slenderness parameter (*α*), thereby accounting for size effects [31]. Measured imperfection value was taken from Ma et al. [1]. The adopted eigenmode inherently captures critical regions of instability, including zones around perforations, and no additional localized imperfection was imposed. An assumed local imperfection value (*δ*_*asd*_) for SHS calculated as:

3 $$\:{\delta\:}_{asd}=\left(\frac{\delta\:}{\alpha\:}\right)avg\:x\:\alpha\:\:\:\:\:\:\:\:\:\:\:\:\:\:\:\:\:\:\:\:\:\:\:\:\:\:\:\:\:\:\:\:\:\:\:\:\:\:\:\:\:\:\:\:\:\:\:\:\:\:\:\:\:\:\:\:\:\:\:\:\:\:\:\:\:\:\:\:\:\:\:\:\:\:\:\:\:\:\:\:\:\:\:$$

where α= *(D-2R)/t*. The δ/α ratio are 0.0119 and 0.0146 for S700 steel grade.

### Finite element modelling

**Fig. 2:**
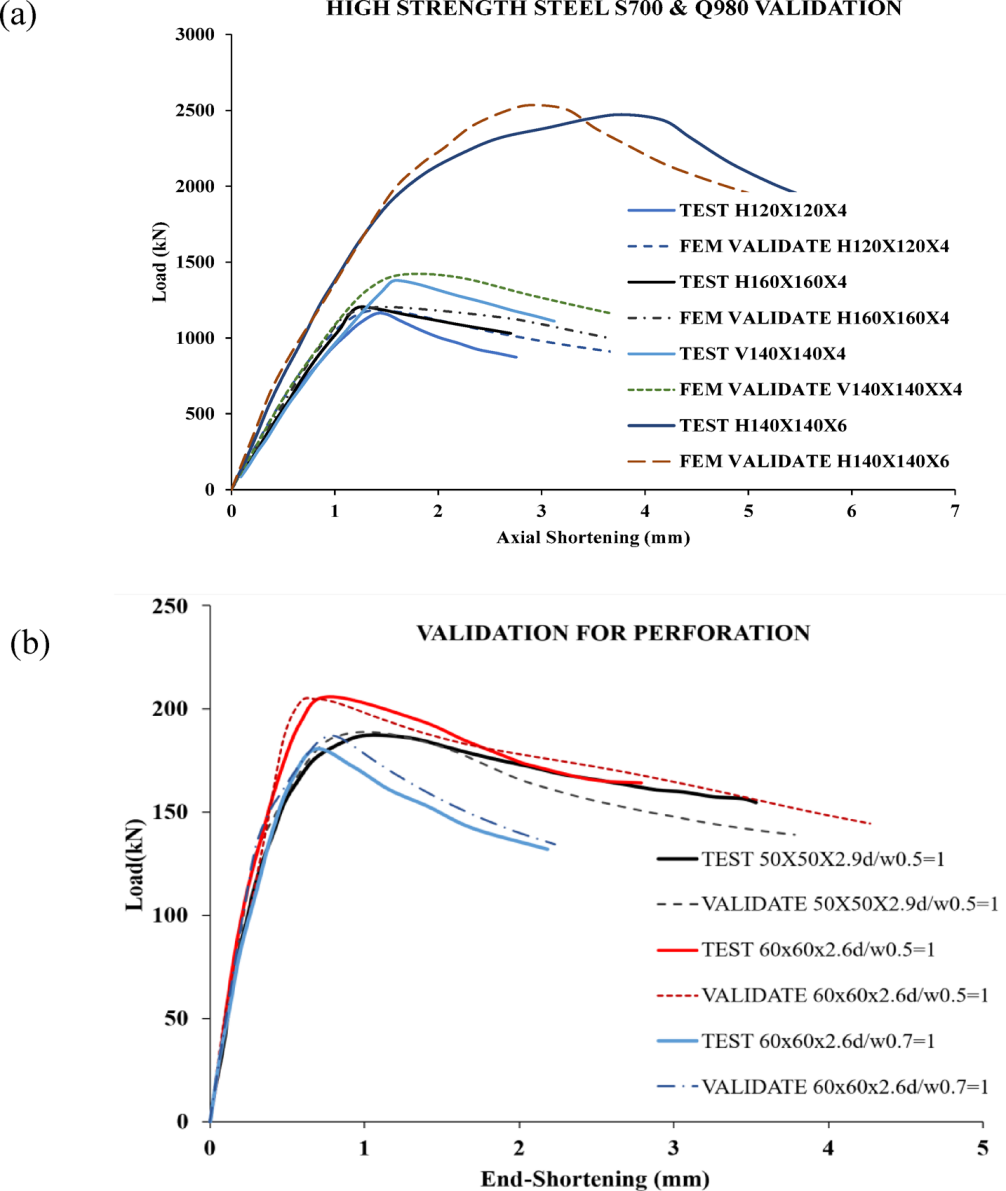
Comparison of load versus axial shortening of test result and present FE model from (**a**) Ma et al.^1^ and (**b**) Singh and Singh^6^.

Square hollow section (SHS) made of cold-formed high-strength steel (CFHSS) of grade S700 was considered in the current study. A schematic diagram of the square hollow cross section along with the designated symbols used in this paper is shown in Fig. 1b. Table 1 provides the cross-section dimensions of the stub column. Length of stub column (*L*) is considered as three times the outer flat width of cross-section (*D*) [2]. Two perforations having varied shapes were considered, one each on opposite sides at the center of SHS stub column. Material property of cold formed high strength steel provided by Ma et al. [1] for flat and corner regions of SHS were adopted in the study. Table 2 provides the basic material properties of HSS S700 derived from flat and corner material coupon tests. The residual stress was neglected in the model as it has negligible effect on the ultimate load (P_u_) and axial shortening [1]. The material property provided by Ma et al. [1] was used by converting it into true stress and true plastic strain [24]. The boundary conditions were assigned using two reference points such as RP-1 (associated with the top end) and RP-2 (associated with the bottom end) by constraining column ends through kinematic coupling shown in Fig. 2. The bottom end (RP-2) was fully restrained against all translational and rotational degrees of freedom, while the top end (RP-1) was restrained against rotation but allowed axial displacement. This configuration ensures uniform load introduction without eccentricity under concentric axial compression. Using displacement control, a central concentrated load was applied statically at the top through reference point RP-1, ensuring uniform loading along the top surfaces. In general, the failure of short column is dominated by localized buckling as shown in Fig. 1c and hence, in the present study, only local geometric imperfections were considered and similar approach has been adopted in several studies [1,8,20,32,39,41]. This imperfection amplitude given in Eq. (3) has been successfully adopted by Ma et al. [2,31] and are found to predict accurate results. The S4R element type for meshing was utilized throughout the model and an optimized mesh size of (B + H)/30 was adopted based on the convergence study from Ma et al. [1]. For perforation, a fan-type meshing proposed by Devi and Singh [23] was utilized in order to get accurate stress contour over the perforation area.

### Validation

A two-step validation scheme was adopted due to lack of experimental data on perforated CFHSS SHS stub column. It is important to note that direct experimental validation for perforated high-strength steel SHS stub columns is currently unavailable in the literature. Therefore, the validation of perforation behavior is based on carbon steel specimens. Nevertheless, the adopted modelling approach captures the fundamental structural response and is considered sufficiently reliable for parametric investigation. General modelling procedures, material input, loading scheme and boundary conditions of CFHSS SHS stub column are validated using test results from Ma et al. [1], which is aided by validation of perforated carbon SHS stub column with test results from Singh and Singh (2018). Figure 2a shows a comparison of test and FE load-deformation curves of CFHSS SHS stub column. A good agreement in the overall load-deformation response has been observed, with minimal deviation of ~ 1.6% and < 3% with respect to the test result for ultimate load and deformation at ultimate load respectively. Additional validation of perforated SHS stub column (having two perforations) is provided in Fig. 2b, however with that of a carbon steel hollow section member due to lack of test data on perforated HSS member. The comparison shows the accuracy of the adopted modelling procedure in predicting the response of perforated hollow section member. Therefore, the adopted modelling procedure detailed in Sect. 2.4 can be considered adequately accurate for perforated HSS tubular column. Consequently, the same has been adopted for the parametric study. A quantitative comparison between experimental [1,6] and FE results is presented in Tables 4 and 5. The mean ratio of test-to-FE capacity is close to unity with low coefficients of variation, indicating good agreement and confirming the reliability of the adopted numerical modelling approach.

**Table 4:** Validation of CFHSS SHS stub columns (Test^1^ vs. FE).

Section	*P*_u, exp_ (kN)	*P*_u, FE_ (kN)	*P*_u, exp_/*P*_u, FE_
H120 × 120 × 4	2380	2445	0.973
H140 × 140 × 4	2450	2515	0.974
H160 × 160 × 4	1200	1235	0.972
V140 × 140 × 6	1350	1390	0.971
**Mean**			**0.973**
**COV (%)**			**0.14**

**Table 5:** Validation of perforated SHS stub columns (Test^6^ vs. FE).

Section	*P*_u, exp_ (kN)	*P*_u, FE_ (kN)	*P*_u, exp_/*P*_u, FE_
50 × 50 × 2.9 d/w 0.5 = 1	180.5	184.2	0.980
60 × 60 × 2.6 d/w 0.5 = 1	205.6	209.8	0.980
60 × 60 × 2.6 d/w 0.7 = 1	135.4	139.1	0.973
**Mean**			**0.980**
**COV (%)**			**0.63**

## Parametric study

By adopting the validated modelling procedure from Sect. 2.5, a parametric study was conducted on perforated CFHSS SHS stub columns with different cross-sections, as listed in Table 6. To investigate the effect of perforation shape, circular, flat-oval, and rectangular openings were compared on an equal-area basis. The characteristic width (or equivalent diameter) of the perforation was maintained constant, while other geometric parameters were adjusted to ensure equal opening area. This ensures that the observed differences in structural response are attributed primarily to shape effects rather than variations in material removal. For the study of perforation height, rectangular openings were considered. The perforation width was kept constant, and the height was varied to achieve different aspect ratios, resulting in a corresponding change in opening area. This approach allows the influence of perforation height to be evaluated independently. The scope of the present study is limited to this arrangement. The aim of the parametric study is as follows-.

**Fig. 3:**
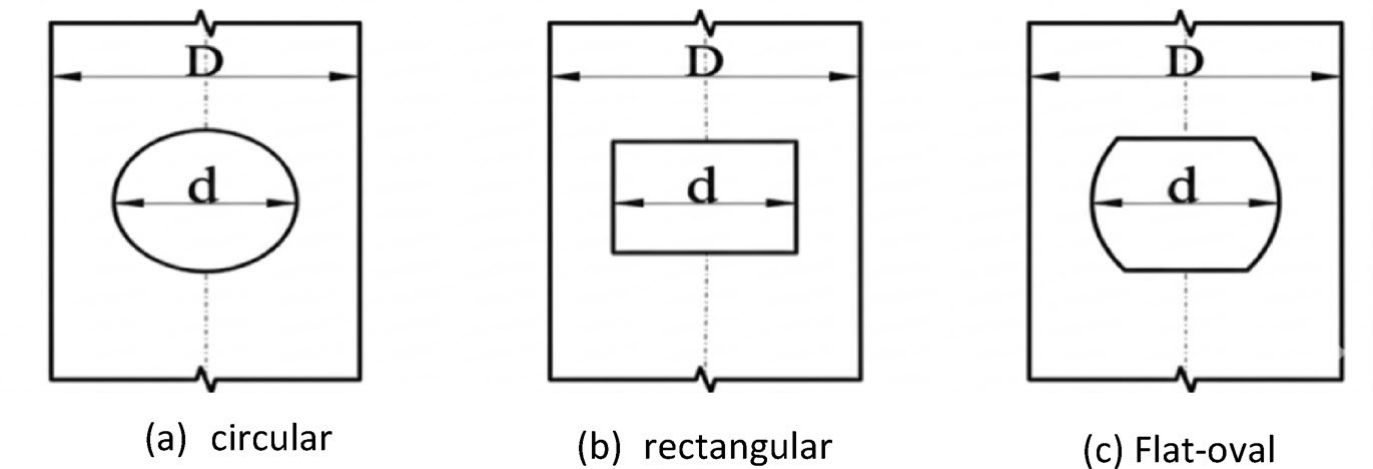
Perforation shapes adopted in the study.

1. To study the size effect on perforation shapes on a CFHSS SHS stub column. The effect of perforation shapes-circular, rectangular and flat-oval (see Fig. 3) has been investigated. Four distinct perforation size ratios of 0.1,0.3,0.5 and 0.7 for perforation hole-width/section width (*d/D*) were analyzed.

2) To study the effect of cut-out height on a rectangular perforated CFHSS SHS stub column for four distinct perforated hole-height to hole-width ratios (*h/d*) of 1,1.5,2 and 2.5 at a constant perforation width/diameter approximately equal to 50% of cross-section width. Also, the length of the column was taken as three times the width of the widest plate element to avoid end effects.

**Table 6:** Cross-section dimensions of perforated square hollow section adopted for parametric studies.

Specimen	Length L (mm)	Width D (mm)	Thickness t (mm)	Depth H (mm)	Outer radius *R* (mm)	d/D (mm)
J1-S-150 × 150	450	150	varies	150	2*t*	varies
J1-S-300 × 300	900	300	varies	300	2*t*	varies
J1-S-70 × 70	210	70	varies	70	2*t*	varies

## Design codes

### General

The effect of perforations on CFHSS SHS stub column is assessed and the proposed axial compressive column capacity (*P*_*ne*_) are then compared with the unfactored design column strength (*P*_*u*_) given in DSM and modified-DSM [27–31,42] for CFHSS SHS stub columns (as in Tables 3 and 4).

### AISI-direct strength method^27^

The strength prediction equations in the DSM consider different buckling modes and the potential interaction between them. For instance, in compression, the DSM includes provisions for local, distortional, and global buckling, each with its own strength curve. For bending and shear, similar relationships are established, allowing for a unified and rational approach across different failure mechanisms. DSM is particularly advantageous for the design of advanced and non-standard sections, such as perforated members, built-up sections, and high-strength steel profiles, where traditional methods may not be accurate or applicable.

The global and local buckling capacity are given by the following formula:

- *Global Buckling (*$$\:{P}_{ne}$$*)*:

4 $$\:{P}_{ne}=\left\{\begin{array}{cc}\left({0.658}^{{\lambda\:}_{g}^{2}}\right){P}_{y}&\:\text{if } {\lambda\:}_{g}\le\:1.5\\\:\left(\frac{0.877}{{\lambda\:}_{g}^{2}}\right){P}_{y}&\:\text{if }{\lambda\:}_{g}>1.5\end{array}\right.$$

where $\:{\lambda\:}_{g}=\sqrt{{P}_{y}/{P}_{cre}}$, $\:{P}_{y}={A}_{g}{F}_{y}$, and $\:{P}_{cre}$ is the critical global buckling load.

b) *Local Buckling (*$$\:{P}_{nl}$$*)*:

5 $${P}_{nl}=\left\{\begin{array}{cc}{P}_{ne}\le\:\:{P}_{ynet}&\:\text{if }{\lambda\:}_{l}\le\:0.776\\\:\left(1-0.15{\left(\frac{{P}_{crl}}{{P}_{ne}}\right)}^{0.4}\right){\left(\frac{{P}_{crl}}{{P}_{ne}}\right)}^{0.4}{P}_{ne}\le\:\:{P}_{ynet}&\:\text{if }{\lambda\:}_{l}>0.776\end{array}\right.$$

where, $\:{\lambda\:}_{l}=\sqrt{{P}_{ne}/{P}_{cr\mathcal{l}}}$; $\:{P}_{ynet}={A}_{net}\mathrm{*}{f}_{y}$; $\:{A}_{net}={A}_{g}-{A}_{p}$; $\:{P}_{cr\mathcal{l}}={A}_{g}\mathrm{*}{f}_{cr\mathcal{l}}$; $\:{A}_{g}$ = gross cross-sectional area; $\:{A}_{p}=2\mathrm{*}d\mathrm{*}t.$

### Modified DSM equation

Apart from the original DSM guideline [27], modified DSM equations proposed by several researchers such as Ma et al. [31], Rossi and Rasmussen [28] and Arrayago and Rasmussen [29,30] are also considered for comparison.

####  Proposed by Ma et al.^31^

Ma et al. [31] proposed a novel empirical equation to predict the normalized axial strength of high-strength steel (HSS) members, focusing particularly on square and rectangular hollow sections (SHS and RHS). The equation presented as Eq. (6), addresses the complex interaction between local buckling and overall column behavior. The motivation behind this equation stems from the observation that traditional design standards that tend to provide conservative estimates when applied to HSS members. These codes were largely calibrated based on tests of mild steel and may not fully capture the nuanced behavior of materials with yield strengths exceeding 460 MPa. HSS exhibits different post-buckling and strain-hardening characteristics, making it imperative to develop tailored design models. Ma et al. [31] proposed a piecewise expression for the which depends on the non-dimensional slenderness parameter.

6 $$\:{P}_{nl}=\left\{\begin{array}{cc}-\left[1-0.09{\left(\frac{1}{\lambda\:}\right)}^{0.9}\right]{\left(\frac{1}{\lambda\:}\right)}^{0.9}+2&\:\text{ (}\text{}{\lambda\:=-\lambda\:}_{l}+1.78\:\mathrm{w}\mathrm{h}\mathrm{e}\mathrm{n}\:{\lambda\:}_{l}\le\:0.89)\\\:\left[1-0.09{\left(\frac{1}{\lambda\:}\right)}^{0.9}\right]{\left(\frac{1}{\lambda\:}\right)}^{0.9}&\:\text{}{(\lambda\:=\lambda\:}_{l}\:\mathrm{w}\mathrm{h}\mathrm{e}\mathrm{n}\:{\lambda\:}_{l}>0.89)\end{array}\right.$$

where $$\:{\lambda\:}_{l}=\sqrt{{P}_{ne}/{P}_{n\mathcal{l}}}$$.

####  Modified DSM equation by Rossi and Rasmussen^28^

Rossi and Rasmussen [28] proposed a modified Direct Strength Method (DSM) formulation to better predict the local buckling strength of tubular columns. This work emerged in response to the growing recognition that conventional DSM equations, originally developed for carbon steel, do not accurately capture the inelastic behavior and strain-hardening effects characteristic of stainless steel. These differences necessitate modifications to the existing DSM framework to enable more accurate and efficient structural design. The proposed Eq. (7) defines the local buckling capacity of stainless-steel columns as a function of the non-dimensional slenderness and the strain-hardening ratio.

7 $$\:{N}_{cl}=\left\{\begin{array}{cc}\left(1-2.11{\lambda\:}_{l}\left(\frac{{\sigma\:}_{u}}{{\sigma\:}_{0.2}}-1\right)+1\right){N}_{y}&\:\text{if }{\lambda\:}_{l}\le\:0.474\\\:\left(\frac{0.95}{{{\lambda\:}_{l}}^{0.8}}\right)-\left(\frac{0.22}{{{\lambda\:}_{l}}^{1.6}}\right){N}_{y}&\:\text{if }{\lambda\:}_{l}>0.474\end{array}\right.$$

where $$\:{\lambda\:}_{l}=\sqrt{{P}_{ne}/{P}_{cr\mathcal{l}}}$$.

####  Modified DSM equation by Arrayago and Rasmussen^29^

Arrayago, and Rasmussen [29] proposed an improved design equation by proposing a modified Direct Strength Method (DSM) that incorporates material nonlinearity and strain-hardening effects. Their work, which builds on earlier research by Rossi and Rasmussen [28], focuses on providing a more accurate prediction of local buckling resistance in tubular steel members, particularly in light of the limitations of traditional DSM approaches that were developed primarily for carbon steel. This formulation given in Eq. (8) enhances the conventional DSM by directly embedding the influence of strain-hardening in the low-slenderness regime. For stocky sections (*λ*_*l*_ *≤ 0.474)*, the first part of the equation captures the increased strength observed in experiments due to the ability of tubular steel to undergo significant plastic deformation before local buckling. For slender sections (*λ*_*l*_ *> 0.474*), the second part provides a rational curve based on regression fits of experimental and numerical data, accounting for the transition to elastic local buckling behavior.

8 $$\:{R}_{en{h}_{nl}}=\left\{\begin{array}{cc}1+\left(1-2.11{\lambda\:}_{l}\left(\frac{{\sigma\:}_{u}}{{\sigma\:}_{0.2}}-1\right)\right){R}_{y}&\:\text{if }{\lambda\:}_{l}\le\:0.474\\\:\left(\frac{0.95}{{{\lambda\:}_{l}}^{0.8}}\right)-\left(\frac{0.22}{{{\lambda\:}_{l}}^{1.6}}\right){R}_{y}&\:\text{if }{\lambda\:}_{l}>0.474\end{array}\right.\:\:\:\:\:\:\:\:\:\:\:\:\:\:\:\:\:\:\:\:\:\:\:\:\:\:\:\:\:\:\:\:\:\:\:\:\:\:\:\:\:\:\:\:\:\:\:\:$$

where, $\:{\sigma\:}_{u}$ is the ultimate tensile strength $\:{\sigma\:}_{0.2}$ is the 0.2% proof (yield) strength; $\:{R}_{en{h}_{nl}}\:$is the normalised yield strength.

### Reliability analysis

Reliability analysis was conducted for proposed equation by the author, direct strength method prediction [27] and other column capacity prediction equation from several researchers [7,24,31]. A design rule was regarded as probabilistically safe if their reliability index *β* is greater than the target value of 2.5, in accordance with the AISI Specification [27] for cold-formed steel members. Previous studies [10,11,23,24] have similarly assessed the adequacy of proposed design models by verifying that the achieved reliability index meets or exceeds this target value. The resistance factor values *ϕ*_*b*_ were found to be different for various design guidelines. The load combinations of 1.2DL + 1.5LL as specified in AS1170 for Australian standards, 1.2DL + 1.6LL for American standards, and 1.35DL + 1.5LL for European standards were adopted in the calculation of reliability index in this paper. A fixed dead-to-live load ratio of 0.2 was used according to the North American specification (AISI 2016 [27]). Other statistical parameters, including mean values and COV, *M*_*m*_=1.1, *F*_*m*_=1.0, *V*_*m*_=0.1, and *V*_*f*_=0.05 were used for material factor and fabrication factor, respectively; *P*_*m*_ and *V*_*p*_ are the corresponding mean value and COV for the set of experimental/FE-to-predicted data. An additional constant, 0.212, accounts for statistical uncertainty, often related to model error or measurement variability. The terms 𝜉 and 𝜙 in the numerator represent the nominal or target resistance and the partial safety factor used in the limit state design approach, respectively. The correction factor *C*_*p*_ was adopted to account for the influence from limited data samples. The equation for calculation of reliability index is shown in Eq. (9).

9 $$\:\:\beta\:=\mathrm{l}\mathrm{n}\left(\frac{{M}_{m}\cdot\:{F}_{m}\cdot\:{P}_{m}}{\xi\:\phi\:}\right)/\sqrt{{V}_{M}^{2}+{V}_{F}^{2}+{V}_{P}^{2}+0.212}$$

**Table 7:** Comparison of FE result with design prediction for stocky & slender sections at hole size *0.5 < d/D < 0.7* and at *d/D < 0.5*.

	*P*_u_/*P*_DSM_		*P*_u_/*P*_DSM−RR_		*P*_u_/*P*_DSM−ARR_		*P*_u_/*P*_DSM−Ma_		*P*_u_/*P*_DSM−Prp_	
	0.5 < d/D < 0.7	d/D < 0.5	0.5 < d/D < 0.7	d/D < 0.5	0.5 < d/D < 0.7	d/D < 0.5	0.5 < d/D < 0.7	d/D < 0.5	0.5 < d/D < 0.7	d/D < 0.5
n	48	49	48	49	48	49	48	49	48	49
Mean	0.59	0.61	0.79	0.93	0.70	0.83	0.58	0.78	1.16	1.19
stdv	0.13	0.2	0.01	0.03	0.02	0.02	0.25	0.04	0.05	0.05
cov	0.22	0.4	0.01	0.03	0.03	0.02	0.44	0.06	0.04	0.04
Vr	0.02	0.1	0.01	0.01	0.01	0.01	0.07	0.01	0.01	0.01
*β*	0.8	0.9	2.2	3.0	1.6	2.4	0.7	2.2	4.1	4.2

## Result and discussion

### Effect of perforation shape and size on column response and P_u_

**Fig. 4:**
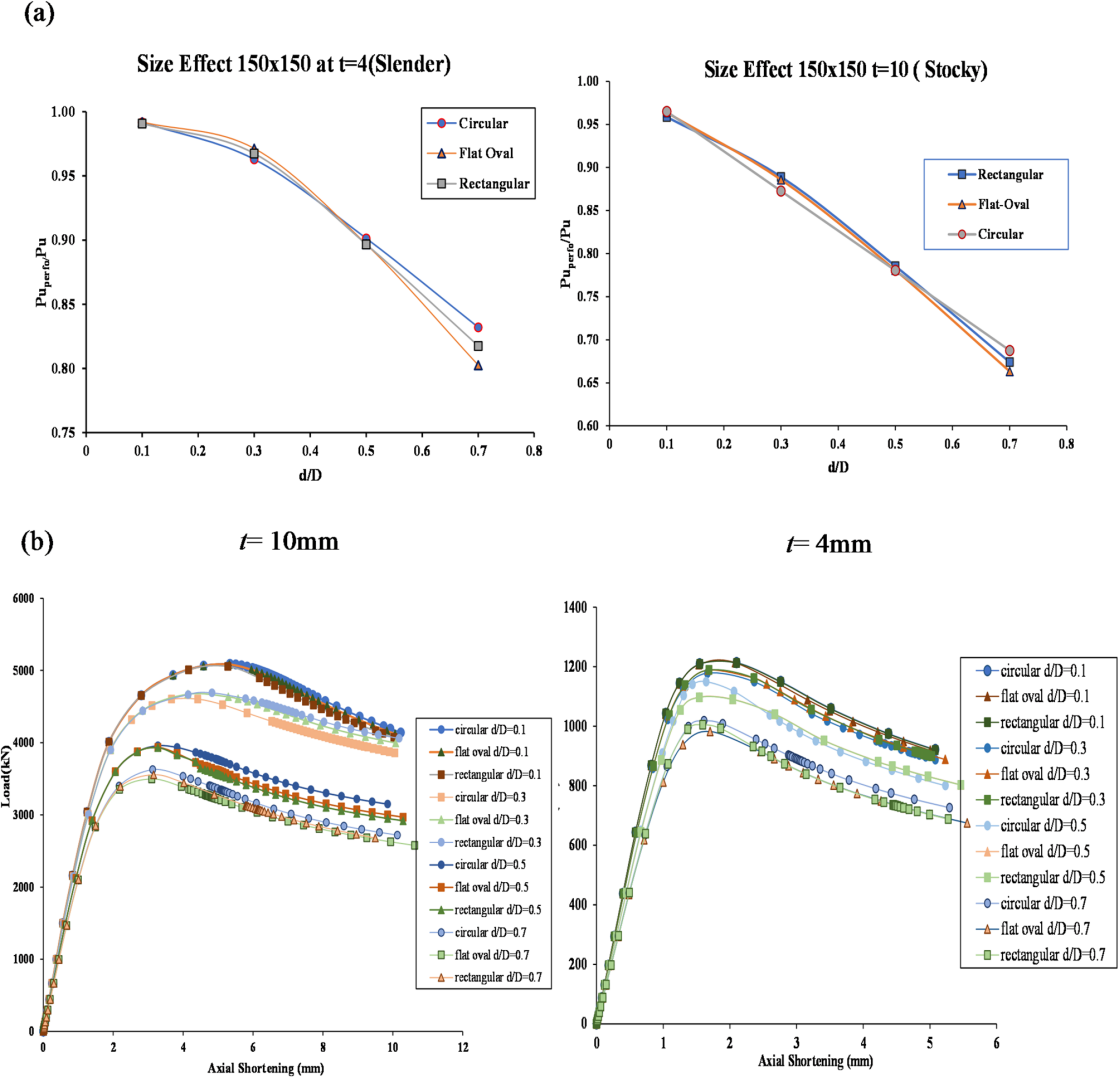
(**a**) Effect of perforation size (*d/D*) and shape on *P*_*uperfo*_*/P*_*u*_ for stocky and slender sections. (**b**) Load–axial shortening curve for SHS (150 mm × 150 mm) columns with different perforation shapes and sizes (*d/D* = 0.1–0.7).

**Fig. 5:**
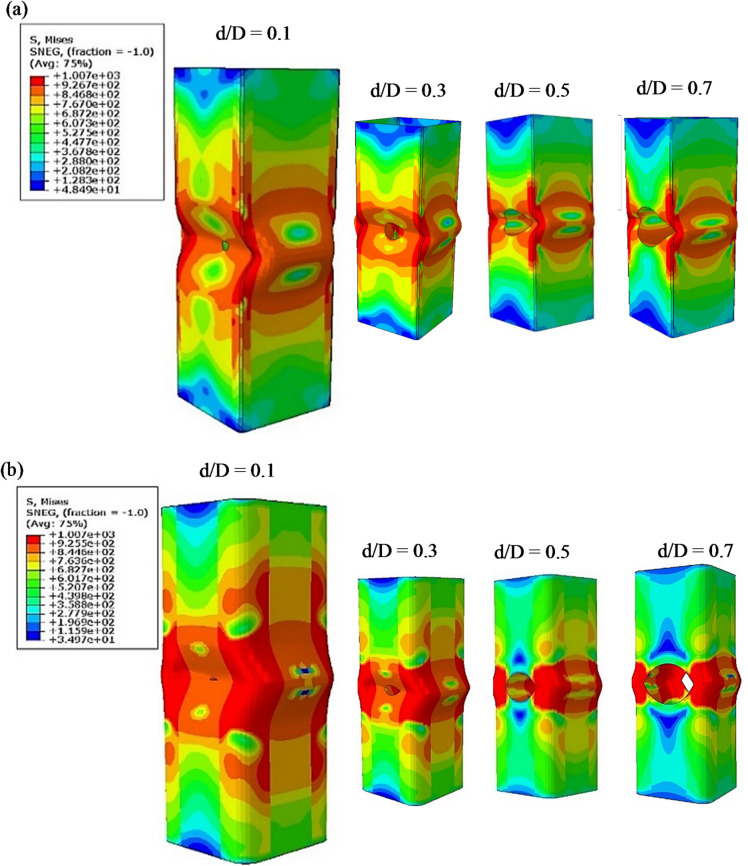
Von Mises stress contour (superimposed with deformed shapes at δ = δu) for circular perforated stub column at *d/D* = 0.1, 0.3, 0.5 and 0.7 with thickness variation: (**a**) *t* = 10 mm (Class 1–3 section) (**b**) *t* = 4 mm (Class 4 section).

In this section, the influence of perforation shape on load-deformation response of perforated CFHSS SHS stub column is discussed. The results from Fig. 4a,b illustrate the effect of perforation size on the axial load capacity (*P*_*u*_) of columns with different perforation shapes—circular, flat-oval, and rectangular (see Fig. 3)—under slender (*t = 4 mm*) and stocky (*t = 10 mm*) conditions. In both cases, as the perforation size increases (represented by a higher *d/D* ratio), the normalized axial capacity (*P*_*uperfo*_*/P*_*u*_) decreases for all column shapes. This trend indicates that larger perforations reduce the column’s ability to carry axial load. For slender columns, the reduction in capacity becomes more pronounced at higher *d/D* values, especially for the flat-oval shape, which shows the steepest decline. The circular section showed a decrease of ~ 15.15%, while the rectangular section decreased by ~ 16.16%. The flat-oval section experienced the highest reduction at about 19.19%, indicating greater sensitivity to perforation in slender members. Circular and rectangular sections exhibit slightly better performance but still show a consistent reduction with increasing *d/D*. For stocky columns, a similar pattern is observed, although the overall drop in capacity is more uniform across all shapes, and the differences between the shapes are less distinct compared to slender columns. The circular section showed a decrease of roughly 28.91%, the rectangular section dropped by 29.31%, and the flat-oval section again experienced the highest reduction at ~ 30.37%. These results highlight that while all shapes are affected by increasing perforation size, flat-oval columns tend to lose a larger proportion of their axial capacity, and stocky columns are generally, more impacted than slender ones in terms of absolute reduction. Figures 5(a) and 5(b) illustrate the von Mises stress distributions for various configurations. In perforated columns, especially at higher *d/D* ratios, stress concentrations develop sharply around the hole boundaries. For instance, specimen (with *d/D* = 0.3) show localized high-stress regions, which become more widespread at with *d/D* = 0.7. While this is still significant, the nature of failure in slender columns is more controlled, governed mainly by plate-buckling. The reduction in load capacity is less abrupt in comparison to stocky columns which is also shown by Singh and Chan [39], and the load-shortening curves remain smoother throughout the deformation range.

The reduction in axial capacity with increasing perforation size can be attributed to the combined effects of local plate buckling and stress concentration around the perforation boundaries. Larger openings disrupt the stress flow and reduce the effective load-carrying area, leading to earlier initiation of local buckling. In non-circular perforations, such as rectangular and flat-oval shapes, sharper edges further intensify stress concentration, accelerating local instability. Mode shape contours further show that perforations reduce stiffness and trigger earlier local buckling near the cut-out. This is evident in circular perforations (see Fig. 5), where d/D increasing results in pronounced deformation concentration around the opening boundary, consistent with previous findings [4,6,24] .

### Effect of perforation height on column response and P_u_

**Fig. 6:**
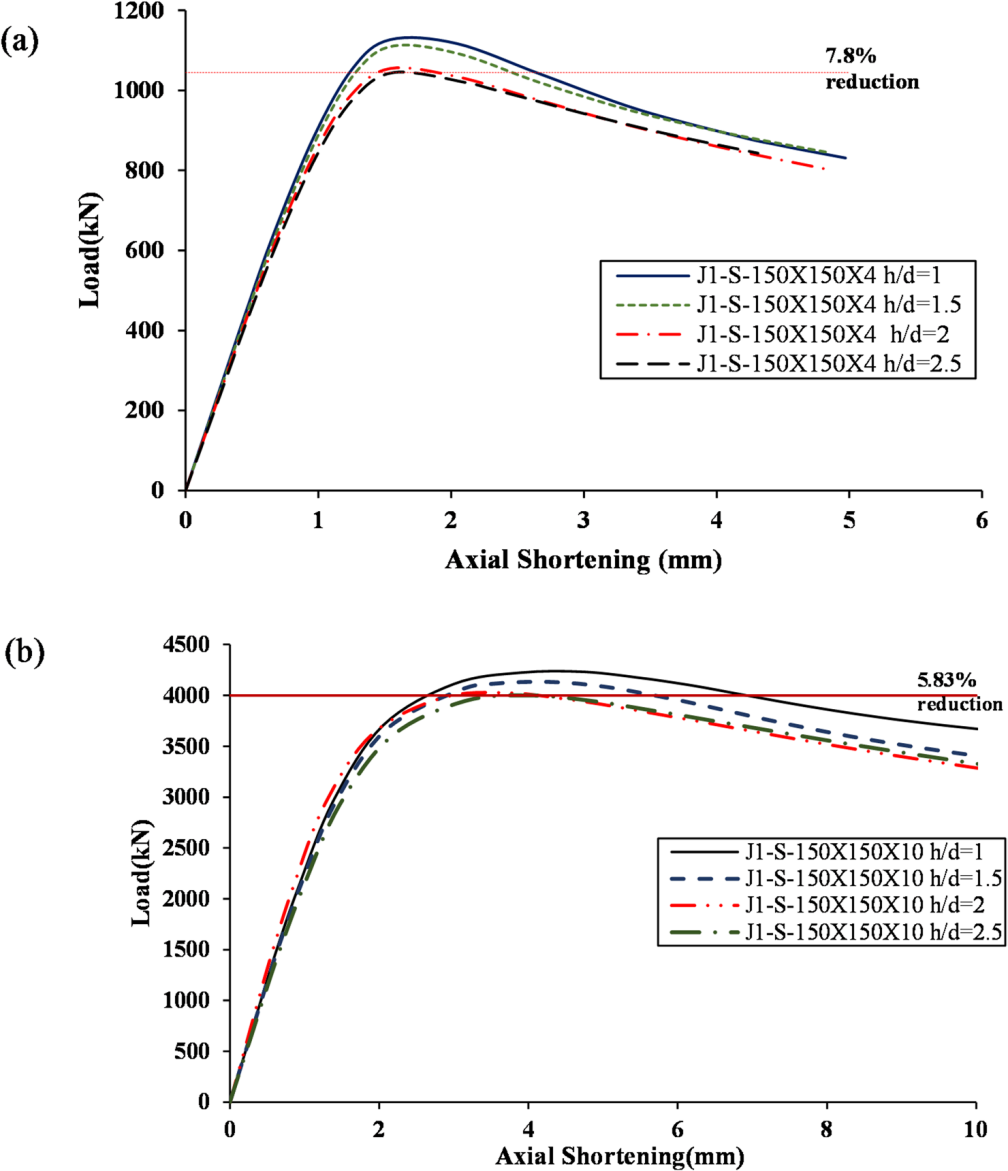
Load versus axial-shortening curves for J1-S-150 × 150 specimen for different values of *h/d* for rectangular perforated (**a**) Slender Column *t* = 4 mm and (**b**) Stocky columns at *t* = 10 mm.

The effect of perforation height on rectangular perforated cold-formed high-strength steel square hollow section (SHS) stub columns can be seen in two separate cases—slender columns (*t* = 4 mm) and stocky columns (*t* = 10 mm). These cases demonstrate how varying the hole height-to-width ratio (*h/d*) of perforations influences the axial load-carrying capacity, represented by the normalized ratio of the ultimate load of perforated to intact columns. For the slender column case (*t* = 4 mm), as the perforation height increases, there is a noticeable decrease in axial load capacity. The axial load-shortening responses shown in Figs. 6(a) and 6(b) plot the axial shortening behavior of slender columns (*t* = 4 mm) with varying *h/d* ratios. The load capacity peaks decrease systematically with increasing h/d, with the *h/d* = 2.5 curve showing the lowest capacity. The reduction in ultimate axial load from *h/d* = 1.0 to *h/d* = 2.5 is marked at approximately 7.8%. Although stocky columns exhibit improved stress redistribution and a more stable post-peak response, the ultimate axial capacity consistently decreases with increasing perforation height. The observed behavior reflects enhanced ductility rather than an increase in strength.

In all sections, it is observed that an increase in the perforation size (*d/D* ratio) leads to a prominent reduction in column capacity. For example, in slender columns with *t* = 4 mm, increasing *d/D* caused a capacity drop of up to ~ 19.19%, while in stocky columns with *t* = 10 mm, the reduction reached over ~ 30%. This effect was more pronounced than those caused by other parameters like perforation shape or height, making size effect a dominant factor.

Unlike perforation shape and height, which may affect stress distribution more locally, perforation size uniformly affects the effective cross-sectional area and the overall column capacity. Larger holes reduce material continuity and introduce higher stress concentrations, which lead to earlier local buckling. Larger perforations result in steeper post-peak declines, sharper stress concentration, and more abrupt failure modes. This consistent degradation supports the need for a reliable design equation on the effect of perforation size. The proposed design curve for perforation size effect is developed using circular perforations as the reference geometry due to their uniform stress distribution and absence of sharp stress concentrations. In this study, all perforation shapes (circular, rectangular, and flat-oval) were analyzed on an equal-area basis to ensure consistent comparison of size effects. Although flat-oval perforations exhibited the greatest reduction in axial capacity in certain cases, the difference relative to circular perforations was found to be limited within the investigated parameter range. Hence, circular perforations provide a rational basis to represent the size effect in a generalized manner. However, it is acknowledged that non-circular perforations may introduce additional stress concentration effects, and the proposed design equation should be applied within the validated parameter range.

### Direct strength method

The predictive performance of the Direct Strength Method (DSM) for perforated SHS stub columns is evaluated by comparing the normalized strength ratios from finite element results with existing DSM formulations. It is observed that the standard DSM provides inconsistent and unsafe (non-conservative) predictions. This behavior arises because the conventional DSM was originally developed for members without perforations and does not inherently account for effects such as stress concentration and reduction in effective load-carrying area. Although provisions for members with holes exist in standards such as AISI S100 [27], their applicability is limited to specific configurations. Therefore, when applied to perforated SHS members, the standard DSM shows reduced prediction accuracy. This limitation highlights the need for a modified design approach that explicitly accounts for perforation size effects.

The decision to propose separate curves for perforation size ratios (*d/D*) up to 0.5 and between 0.5 and 0.7 arises from the observed differences in structural behavior and strength degradation patterns within these two ranges. For *d/D* ≤ 0.5, the reduction in axial strength is moderate and relatively uniform, allowing a single predictive curve to capture the trend accurately while still maintaining conservatism. However, as the perforation size increases beyond 0.5, the influence on the load-carrying capacity becomes more pronounced due to localized buckling effects [39]. In the 0.5 < *d/D* < 0.7 range, this accelerated degradation in performance necessitates a distinct curve that reflects the sharper decline in strength. Grouping all perforation sizes under a single curve would risk either overestimating capacity in the higher range or being excessively conservative for the lower range. Therefore, separating the two allows for more realistic yet conservative modelling, ensuring that the design remains both safe and representative of actual structural behavior across different perforation sizes. At *d/D* > 0.7, the remaining net section of the column is significantly reduced, leading to severe compromise in structural integrity. Additionally, as stated by Singh and Singh [39] perforation size ratio *d/D* beyond 0.7 will also affect the corner curve region beyond the flat width which could result in different post-buckling failure pattern.

#### Perforation with d/D ≤ 0.5

**Fig. 7:**
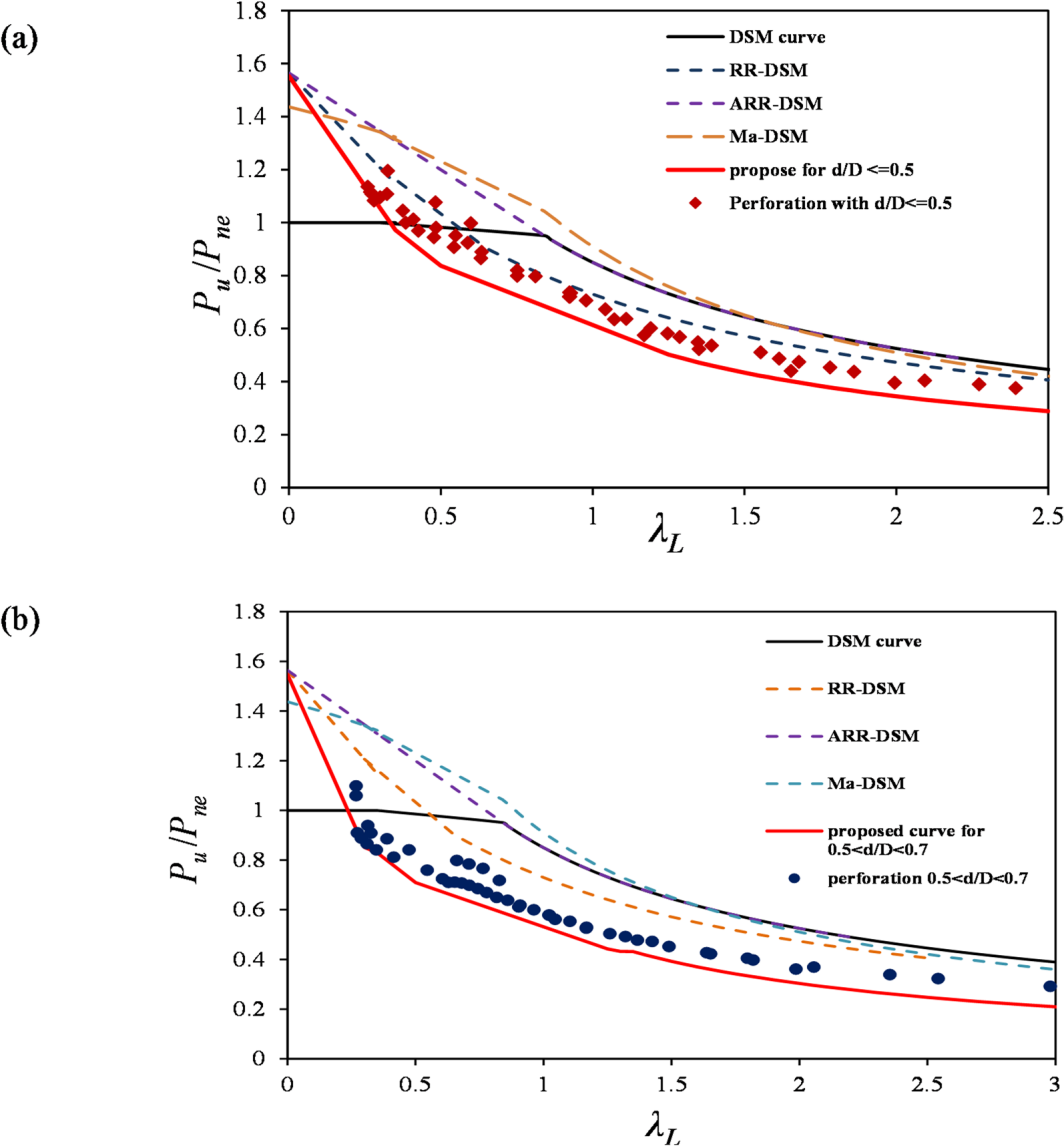
Comparison of FE results with DSM^27–29^ design curve for perforation size ratio (**a**) d/D ≤ 0.5 and (**b**) 0.5 < d/D < 0.7.

Figure 7a, b compare normalized ultimate strength with predictions from various Direct Strength Method (DSM) formulations, where *P*_*u*_*/P*_*ne*_ is the ratio of ultimate axial compressive load to the proposed column capacity and *λ*_*l*_ represents plate slenderness, demonstrating accuracy across different perforation size ratios. These include the standard DSM [27], the modified DSM by Rossi and Rasmussen [28], the adjusted rational regression DSM proposed by Arrayago and Rasmussen [29], Ma et al. [31], and a newly proposed method specifically developed for perforated members. The red solid line represents the proposed method for members with *d/D* ≤ 0.5. The original DSM exhibits a lower mean of 0.61 and a significantly higher coefficient of variation (cov = 0.40), suggesting inconsistent and generally non-conservative predictions as shown in Table 7. Its reliability index (*β* = 0.9) reflects a high risk of unsafe design outcomes when used for perforated members. RR_− DSM,_ ARR-_DSM_ and Ma-_DSM_ show notable improvements (*β* = 3 for RR_DSM_, *β* = 2.4 for ARR_− DSM_ and *β* = 2.2 for Ma-_DSM_) and suggest moderate reliability, making them safer alternatives to the standard DSM but less reliable than the proposed method. In contrast, the proposed tri-linear provides consistently conservative predictions that remain close to the actual performance, ensuring both safety and reliability in design. More notably, the proposed method exhibits the highest reliability index (*β* = 4.2), indicating an improved reliability compared to existing methods within the investigated dataset. The newly proposed design curve, with its formulation as shown in Eq. 10 following equation proposed by Rossi and Rasmussen [28] for perforated columns, fills this gap effectively.

10 $$\:{p}_{u}=\left\{\begin{array}{c}1+1.5\left(1-3{\lambda\:}_{l}\left(\frac{{\sigma\:}_{u}}{{\sigma\:}_{0.2}}-0.8\right)\right){P}_{prp}\:\:\:\:\:\:\:\:\:\:\:\mathrm{i}\mathrm{f}\text{}{\lambda\:}_{l}\le\:0.4\:\:\\\:\left(\frac{1.5}{{{\lambda\:}_{l}}^{0.78}}\right)-\left(\frac{0.9}{{{\lambda\:}_{l}}^{0.9}}\right){P}_{prp}\:\:\:\:\:\:\:\:\:\:\:\:\:\:\:\:\:\:\:\:\:\:\:\:\:\:\:\:\:\:\:\mathrm{i}\mathrm{f}\text{}{0.4\le\:\lambda\:}_{l}\le\:1.2\\\:\left(\frac{1.5}{{{\lambda\:}_{l}}^{0.8}}\right)-\:\left(\frac{0.9}{{{\lambda\:}_{l}}^{0.8}}\right){P}_{prp}\:\:\:\:\:\:\:\:\:\:\:\:\:\:\:\:\:\:\:\:\:\:\:\:\:\:\:\mathrm{i}\mathrm{f}\text{}{\lambda\:}_{l}>1.2\:\:\:\:\:\end{array}\right.$$

where *P*_*u*_ is the predicted axial compressive capacity, *P*_*prp*_ is the proposed reference axial capacity of the perforated member, λl is the local slenderness parameter, *σ*_*u*_ is the ultimate tensile strength, and *σ*_*0.2*_ is the 0.2% proof stress of the material.

#### Perforation with 0.5 < d/D < 0.7

Another hole size ratio *d/D* within the range of 0.5 < *d/D* < 0.7 is also proposed. The proposed model achieves the highest reliability index of 4.1 (see Table 7), significantly exceeding the threshold values commonly recommended for structural design applications. This suggests a low probability of failure and robust performance under uncertain loading conditions. On the other hand, the DSM and Ma-_DSM_ yield *β* values of 0.8 and 0.7 respectively, which fall short of acceptable safety margins, emphasizing their inadequacy for members with intermediate web perforations. The RR-_DSM_ and ARR_− DSM_ show moderate reliability with *β* values of 2.2 and 1.6 respectively, positioning them as transitional improvements over the conventional DSM but still inferior to the proposed formulation. The proposed method demonstrates superior performance with a mean *P*_*u*_*/P*_*DSM*_ ratio of 1.16, indicating a consistent underestimation of strength by approximately 16%, which is considered safe and acceptable in design contexts. A proposed equation as shown in Eq. 11 following tri-linear model proposed by Rossi and Rasmussen [28] is shown. This equation captures the significance influence of strain hardening contribution to the column capacity making it more reliable to practice behavior of high strength steel stub column.

11 $$\:{p}_{u}=\left\{\begin{array}{c}4.2\left(1-1.5{\lambda\:}_{l}\left(\frac{{\sigma\:}_{u}}{{\sigma\:}_{0.2}}-0.8\right)\right){P}_{prp}\:\:\:\:\:\:\:\:\:\:\:\:\:\mathrm{i}\mathrm{f}\text{}{\lambda\:}_{l}\le\:0.3\:\:\\\:\left(\frac{1.5}{{{\lambda\:}_{l}}^{0.78}}\right)-\left(\frac{1}{{{\lambda\:}_{l}}^{0.9}}\right){P}_{prp}\:\:\:\:\:\:\:\:\:\:\:\:\:\:\:\:\:\:\:\:\:\:\:\:\:\:\:\:\:\:\:\mathrm{i}\mathrm{f}\text{}{0.3\le\:\lambda\:}_{l}\le\:1.3\\\:\left(0.75\frac{0.95}{{{\lambda\:}_{l}}^{0.8}}\right)-\:\left(\frac{0.2}{{{\lambda\:}_{l}}^{0.5}}\right){P}_{prp}\:\:\:\:\:\:\:\:\:\:\:\:\:\:\:\:\:\:\:\:\:\:\:\:\:\:\:\mathrm{i}\mathrm{f}\text{}{\lambda\:}_{l}>1.3\:\:\:\:\:\end{array}\right.$$

The proposed equations are applicable to cold-formed high-strength steel square hollow section stub columns subjected to concentric axial compression. The validity is limited to the investigated range of parameters, including perforation size ratio up to *d/D* = 0.7, the local slenderness range (*λ*_*l*_), double-sided symmetric perforations, and stub column behavior (L ≈ 3D). Extension beyond these ranges requires further validation.

## Conclusion

This study investigated the structural performance of CFHSS SHS stub columns featuring centrally located web perforations arranged in a double-sided symmetrical configuration of varying shapes, sizes, and geometrical ratios. Through detailed finite element analysis, the research focused on how perforation shape, plate thickness, *d/D* (hole width to section width) ratio, and *h/d* (perforation height to width) ratio influence the ultimate load capacity (*P*_*u*_) and load-deformation behavior of both slender and stocky column sections. The results were compared against various Direct Strength Method (DSM) formulations [27–29], leading to the development of a new, more reliable design model for perforated members. The following key findings and conclusions drawn from the numerical investigation are presented below:

1. Strain-hardening behavior was significantly more pronounced in thicker columns (*t* ≥ 10 mm) from the adopted dimensions and larger sections (e.g., 300 × 300 mm), contributing to improved post-peak load retention. In contrast, thin-walled slender columns (e.g., 2 mm thickness) exhibited minimal to no strain-hardening and failed quickly after reaching peak load, primarily due to plate-buckling and low plastic reserve.
2. Increasing the *d/D* ratio from 0.1 to 0.7 caused *P*_*u*_ reductions of up to 34.4% in stocky columns and 29.4% in slender columns due to size effect. Flat-oval openings showed the most sensitivity, especially in slender members with up to 19.19% reduction compared to 15.15% (circular) and 16.16% (rectangular). Stocky columns showed more uniform reduction but suffered steeper post-peak declines, indicating a brittle failure pattern at high *d/D* values.
3. In the investigation of height effect, increasing *h/d* from 1.0 to 2.5 reduced Pu by ~ 7.8% in slender columns, with more severe stress concentration effects and earlier local buckling. Stocky columns showed better resilience, maintaining *P*_*u*_ above that of intact members across all *h/d* values, likely due to their enhanced confinement and reduced slenderness.
4. The standard DSM significantly overpredicts the strength (mean = 0.61, *β* = 0.9), posing a risk of unsafe design for perforated members. The proposed tri-linear model with respect to size effect for *d/D ≤ 0.5 and 0.5 < d/D < 0.7* achieved a higher reliability index (*β* = 4.1, 4.2) and conservative mean prediction (*Pu/P*_*DSM*_ = 1.15–1.16) with low variability (cov ≈ 0.04), demonstrating comparatively better performance among the considered methods. This method effectively accounts for strain-hardening and geometric discontinuities, offering a safer and more accurate design approach for HSS stub columns with perforation.

## Limitations of the study

The study is confined to short (stub) columns, with a primary focus on the effect of perforations; therefore, other influencing parameters, such as variations in column length and thickness, are not considered.

## Supplementary Information

Below is the link to the electronic supplementary material.

Supplementary Material 1

## Data Availability

The datasets generated and or/ analysed during the current study are not publicly available resulting in more length of the paper (the end results are given), but are available from the corresponding author on reasonable request.

## Abbreviations

HSS: High-strength steel
SHS: Square hollow section
CFS: Cold-formed steel
FEM: Finite element method
RR-DSM: Modified DSM (Rossi and Rasmussen)
Ma-DSM: Modified DSM (Ma et al.)
Pu: Ultimate axial load
Pprp: Proposed axial compressive capacity
Pynet: Yield load (net section)
Anet: Net cross-sectional area
*fy*: Yield strength
*λl*: Local slenderness parameter
*h*/*d*: Perforation height to width ratio
*L*: Column length
*t*: Thickness
*σ*_0.2_: 0.2% proof stress
*εf*: Fracture strain
υ: Poisson’s ratio
COV: Coefficient of variation
*d*: Perforation width
CFHSS: Cold-formed high-strength steel
RHS: Rectangular hollow section
FE: Finite element
DSM: Direct strength method
ARR-DSM: Modified DSM (Arrayago and Rasmussen)
AISI: American iron and steel institute
Pne: Nominal axial compressive strength
Pcrl: Elastic local buckling load
Ag: Gross cross-sectional area
Ap: Area removed due to perforation
*E*: Young’s modulus
*d*/*D*: Perforation size ratio
*D*: Section width
*R*: Corner radius
*σ*_u_: Ultimate tensile strength
*n*: Strain hardening exponent
*β*: Reliability index
