# Supplementary material for: Axial compression behavior and design of perforated high-strength steel square hollow section stub columns
Source: Sci Rep. 2026 May 18;16:22589. doi: 10.1038/s41598-026-53799-4 (PMC13381956; doi:10.1038/s41598-026-53799-4)
Supplement: Supplementary file 1 — Supplementary Material 1 [file 41598_2026_53799_MOESM1_ESM.pdf]

RELIABILITYd/D<0.5

CIRCULAR PERFORATION

	Pu	Pv	Pv-RR	Pu/Pv	Pu/Pv-RR	Pu/Pv-ARR	Pu/Pv,P	Pu/Pv-ma	Pv-ARR	Pv,Prp	PDSM,Ma
J1-S-70X70X2	**281.28**	383.7	292.0	0.73	0.96	0.82	1.18	0.76	342.64	238.81	370.12
J1-S-70X70X3.2	**593.17**	595.8	555.6	1.00	1.07	0.96	1.29	0.83	618.71	459.49	716.80
J1-S-70X70X4	**783.87**	729.6	726.8	1.07	1.08	1.01	1.27	0.85	776.06	618.65	920.85
J1-S-70X70X6	**1237.8**	1037.6	1092.7	1.19	1.13	1.09	1.18	0.90	1139.13	1049.91	1378.73
J1-S-300x300x5.4	**2303.2**	4521.2	2528.5	0.51	0.91	0.81	1.21	0.81	2843.95	1902.57	2850.63
J1-S-300x300x2	**410**	1706.7	473.9	0.24	0.87	0.80	1.27	0.90	512.73	322.07	453.25
J1-S-300x300x6	**2677.6**	5006.5	3002.3	0.53	0.89	0.79	1.16	0.77	3401.03	2300.05	3460.90
J1-S-300x300x7	**3490.6**	5807.8	3832.4	0.60	0.91	0.79	1.10	0.76	4392.58	3166.33	4570.18
J1-S-300x300x8	**4434.5**	6599.6	4710.2	0.67	0.94	0.81	1.15	0.76	5463.14	3866.67	5797.82
J1-S-300x300x9	**5424.6**	7382.0	5629.6	0.73	0.96	0.82	1.18	0.76	6610.01	4604.29	7144.58
J1-S-300x300x8.5	**4925.4**	6992.0	5169.2	0.70	0.95	0.82	1.16	0.76	6032.83	4234.08	6463.69
J1-S-300x300x7.5	**3945.2**	6204.9	4267.0	0.64	0.92	0.80	1.12	0.76	4919.80	3512.68	5171.29
J1-S-300x300x6.5	**3068.9**	5408.3	3405.4	0.57	0.90	0.79	1.16	0.77	3879.52	2645.99	3991.79
J1-S-300x300x5	**1986.7**	4195.7	2230.5	0.47	0.89	0.80	1.20	0.80	2497.24	1658.75	2476.79
J1-S-300x300x4.5	**1654.2**	3786.8	1881.1	0.44	0.88	0.79	1.20	0.81	2094.54	1379.34	2049.34
J1-S-300x300x4	**1363.9**	3375.5	1548.3	0.40	0.88	0.80	1.22	0.83	1714.23	1118.77	1651.68
J1-S-300x300x3.5	**1112.2**	2961.8	1239.0	0.38	0.90	0.82	1.26	0.86	1364.06	882.14	1292.12
J1-S-300x300x3.7	**1217.8**	3127.6	1356.1	0.39	0.90	0.81	1.25	0.85	1496.15	970.87	1426.54
J1-S-300x300x4.2	**1400.3**	3540.3	1678.4	0.40	0.83	0.75	1.15	0.78	1862.54	1219.94	1805.90
J1-S-300x300x4.7	**1790.6**	3950.7	2020.2	0.45	0.89	0.79	1.20	0.81	2254.52	1489.99	2218.57
J1-S-300x300x5.2	**2117.4**	4358.6	2378.4	0.49	0.89	0.79	1.19	0.80	2669.02	1779.22	2661.46
J1-S-300x300x6.2	**2829.9**	5167.5	3163.6	0.55	0.89	0.79	1.16	0.77	3592.17	2437.84	3672.42
J1-S-300x300x6.7	**3235.1**	5568.4	3574.4	0.58	0.91	0.79	1.16	0.77	4081.92	2794.03	4219.03
J1-S-300x300x11	**7303.3**	8918.3	7548.9	0.82	0.97	0.81	1.18	0.74	8964.22	6170.63	9877.06
J1-S-300x300x13	**9246.6**	10416.9	9499.6	0.89	0.97	0.86	1.18	0.75	10736.84	7820.41	12320.55
J1-S-300x300x14	**10275**	11151.9	10467.5	0.92	0.98	0.89	1.19	0.76	11606.42	8669.49	13479.84
J1-S-300x300x15	**11275**	11877.5	11418.7	0.95	0.99	0.90	1.18	0.77	12464.33	9529.20	14606.73

J1-S-300x300x17	**13011**	13300.3	13237.5	0.98	0.98	0.92	1.16	0.78	14142.13	11258.67	16773.94
J1-S-70X70X2	**275.74**	383.7	292.9	0.72	0.94	0.80	1.15	0.74	343.99	239.55	371.91
J1-S-70X70X8	**1481.4**	1307.8	1407.9	1.13	1.05	1.02	1.01	0.84	1454.53	1465.93	1769.16
J1-S-70X70X7.3	**1345.2**	1217.5	1305.1	1.10	1.03	1.00	1.01	0.82	1350.68	1338.44	1641.43
J1-S-70X70X7	**1272.3**	1177.4	1258.5	1.08	1.01	0.98	1.00	0.80	1304.01	1277.74	1583.75
J1-S-70X70X6.5	**1211.6**	1108.7	1177.2	1.09	1.03	0.99	1.04	0.82	1223.11	1166.82	1483.26
J1-S-70X70X6	**1147.2**	1037.6	1093.5	1.11	1.05	1.01	1.09	0.83	1139.64	1053.63	1379.43
J1-S-70X70X5.5	**1005.5**	964.1	998.4	1.04	1.01	0.96	1.12	0.80	1048.14	896.73	1262.14
J1-S-70X70X5	**885.84**	888.3	916.9	1.00	0.97	0.92	1.09	0.76	963.87	812.20	1159.44
J1-S-70X70X4.5	**783.53**	810.1	824.2	0.97	0.95	0.90	1.08	0.75	871.71	722.82	1043.13
J1-S-70X70X4	**687.29**	729.6	728.4	0.94	0.94	0.88	1.11	0.75	776.80	621.13	922.36
J1-S-70X70X3.5	**585.7**	646.7	623.4	0.91	0.94	0.86	1.12	0.74	679.28	520.82	796.83
J1-S-70X70X3	**484.95**	561.4	512.6	0.86	0.95	0.84	1.15	0.73	578.87	422.08	664.63
J1-S-70X70X2.5	**378.02**	473.7	401.2	0.80	0.94	0.79	1.15	0.72	476.24	327.93	524.89
J1-S-70X70X2	**275.74**	383.7	292.3	0.72	0.94	0.80	1.15	0.74	343.19	239.11	370.84
J1-S-70X70X2.3	**348.68**	438.0	357.3	0.80	0.98	0.82	1.19	0.75	426.07	291.84	465.88
J1-S-70X70X1.7	**208.25**	328.6	230.8	0.63	0.90	0.78	1.10	0.74	266.97	189.67	282.14
J1-S-70X70X1.5	**167.26**	291.3	194.4	0.57	0.86	0.75	1.04	0.72	223.12	160.43	232.76
J1-S-70X70X1.3	**132.5**	253.7	155.1	0.52	0.85	0.75	1.11	0.74	176.06	119.45	179.93
J1-S-70X70X1	**86.4**	196.6	105.6	0.44	0.82	0.73	1.10	0.73	118.31	78.70	117.59

	Pu/P_{DSM}	Pu/P_{DSM-RR}	$Pu/P_{DSM-ARR}$	Pu/P_{DSM-Ma}	$Pu/P_{DSM-Prp}$
**n**	49	49	49	49	49
**mean**	0.61	0.93	0.83	0.78	1.19
**stdv**	0.2	0.03	0.02	0.04	0.05
**cov**	0.4	0.03	0.02	0.06	0.04
**Vr**	0.1	0.01	0.01	0.01	0.01
**β**	0.9	3.0	2.4	2.2	4.2

RELIABILITY0.7>d/D>0.5

CIRCULAR PERFORATION

	Pu	Pv	Pv-RR	Pu/Pv	Pu/Pv-RR	Pu/Pv-ARR	Pu/Pv-ma	Pu/Pv,P	Pv-ARR	Pv,Prp	PDSM,Ma
J1-S-300x300x8	3807.8	6599.61	4762.0587	0.58	0.80	0.69	0.48	1.17	5533.5467	3253.28	8006.6145
J1-S-300x300x4	1243.7	3375.5	1567.1844	0.37	0.79	0.72	0.28	1.25	1736.5833	995.13	4424.6172
J1-S-300x300x3.5	1001.5	2961.85	1253.541	0.34	0.80	0.73	0.26	1.30	1380.9981	772.56	3918.23
J1-S-300x300x4.5	1502.2	3786.8	1909.3842	0.40	0.79	0.71	0.31	1.20	2128.509	1247.11	4919.4355
J1-S-300x300x5	1790.3	4195.72	2267.8434	0.43	0.79	0.70	0.33	1.18	2542.803	1519.16	5396.5828
J1-S-300x300x5.5	2077.9	4602.29	2642.5352	0.45	0.79	0.70	0.35	1.15	2979.8833	1812.06	5868.6721
J1-S-300x300X6	2390	5006.48	3036.6592	0.48	0.79	0.69	0.38	1.12	3444.332	2130.00	6324.3863
J1-S-300x300X6.5	2720.1	5408.31	3450.2642	0.50	0.79	0.69	0.40	1.14	3937.0744	2375.60	6766.075
J1-S-300x300X7	3066.9	5807.78	3875.8157	0.53	0.79	0.69	0.43	1.15	4449.2623	2659.39	7193.7381
J1-S-300x300X7.5	3429.7	6204.88	4282.4025	0.55	0.80	0.69	0.45	1.17	4940.303	2932.02	7607.3217
J1-S-300x300X8	3807.3	6599.61	4757.544	0.58	0.80	0.69	0.48	1.17	5527.3955	3250.31	8006.9051
J1-S-300x300X8.5	4194.1	7010.61	5229.4996	0.60	0.80	0.69	0.50	1.18	6113.4767	3568.87	8423.5291
J1-S-300x300X9	4578.4	7420.37	5709.6972	0.62	0.80	0.68	0.52	1.18	6716.478	3894.94	8837.3528
J1-S-300x300X9.5	4987.4	7828.89	6200.942	0.64	0.80	0.68	0.54	1.18	7341.163	4230.87	9232.7683
J1-S-300x300X10	5336.2	8236.17	6694.1761	0.65	0.80	0.67	0.55	1.17	7975.1075	4570.81	9659.8458
J1-S-300x300X10.5	5767.4	8642.22	7198.3835	0.67	0.80	0.67	0.57	1.17	8632.3662	4921.52	10033.796
J1-S-300x300X11	6186.5	9047.03	7701.4406	0.68	0.80	0.68	0.59	1.17	9111.3111	5274.71	10410.56
J1-S-300x300X11.5	6590.2	9450.61	8212.7332	0.70	0.80	0.69	0.61	1.17	9584.8106	5637.74	10793.047
J1-S-300x300X12	6957.1	9852.95	8722.6199	0.71	0.80	0.69	0.62	1.16	10055.397	6003.79	11196.145
J1-S-300x300X12.5	7273.6	10254	9237.221	0.71	0.79	0.69	0.63	1.14	10525.867	6378.15	11629.802
J1-S-300x300X13	7544.5	10653.9	9749.1657	0.71	0.77	0.69	0.62	1.12	10993.766	6755.32	12091.504
J1-S-300x300X13.5	7992.6	11052.5	10259.266	0.72	0.78	0.70	0.64	1.12	11459.78	7136.30	12433.274
J1-S-300x300X15	9272.8	12241	11781.787	0.76	0.79	0.72	0.69	1.12	12851.057	8310.40	13477.649
J1-S-300x300X20	13052	16122.2	16463.831	0.81	0.79	0.75	0.76	1.05	17385.466	12412.18	17121.353
J1-S-300x300X25	16683	19879.7	20775.583	0.84	0.80	0.77	0.81	1.01	21727.565	16490.00	20635.526
J1-S-300x300X30	20312	23513.5	24879.279	0.86	0.82	0.78	0.85	1.00	25886.315	20382.33	23913.901
J1-S-300x300X35	23935	27023.6	28809.722	0.89	0.83	0.80	0.89	1.01	29882.994	23666.37	26958.138
J1-S-300x300X40	27569	30410.1	32586.601	0.91	0.85	0.82	0.93	0.99	33729.639	27712.43	29741.904
J1-S-70X70X4	612	729.584	729.4644	0.84	0.84	0.79	0.81	1.16	777.28905	528.96	757.92129

J1-S-70X70X2	**234.27**	383.716	296.07199	0.61	0.79	0.67	0.51	1.16	348.4813	201.97	458.41349
J1-S-70X70X1.7	**184.22**	328.571	233.9471	0.56	0.79	0.68	0.46	1.15	271.23418	159.92	401.47662
J1-S-70X70X1.5	**152.98**	291.335	194.5733	0.53	0.79	0.68	0.42	1.15	223.3858	133.50	361.29698
J1-S-70X70X1.3	**124.63**	253.721	157.2841	0.49	0.79	0.70	0.39	1.12	178.8485	111.37	318.85349
J1-S-70X70X0.5	**29**	99.4772	35.643494	0.29	0.81	0.74	0.22	1.39	38.934274	20.89	133.36574
J1-S-70X70X0.8	**57.048**	158.028	75.106692	0.36	0.76	0.68	0.27	1.19	83.359601	48.07	207.62166
J1-S-70X70X1	**82.89**	196.589	105.59139	0.42	0.79	0.70	0.33	1.17	118.32762	70.57	253.29155
J1-S-70X70X0.9	**71.73**	177.356	90.159245	0.40	0.80	0.71	0.31	1.21	100.5707	59.06	229.8248
J1-S-70X70X0.6	**38.36**	119.089	47.762581	0.32	0.80	0.73	0.24	1.33	52.46159	28.95	158.28474
J1-S-70X70X1.2	**110.67**	234.771	138.7339	0.47	0.80	0.71	0.37	1.15	156.91038	96.25	297.23239
J1-S-70X70X2.2	**301.16**	420.006	339.16941	0.72	0.89	0.75	0.63	1.30	403.40987	231.54	474.28261
J1-S-70X70X2.4	**348.77**	455.917	383.09047	0.76	0.91	0.76	0.70	1.33	457.10352	262.09	499.64634
J1-S-70X70X2.6	**384.51**	491.45	427.50612	0.78	0.90	0.77	0.72	1.31	498.59774	293.52	532.30284
J1-S-70X70X2.8	**419.36**	526.605	472.06723	0.80	0.89	0.78	0.74	1.29	539.68095	325.66	564.83947
J1-S-70X70X7	**1103.2**	1177.42	1244.7458	0.94	0.89	0.85	0.98	1.08	1295.5864	1017.67	1122.5117
J1-S-70X70X6	**940.29**	1037.6	1092.381	0.91	0.86	0.83	0.93	1.06	1138.9538	883.43	1016.2317
J1-S-70X70X5	**784.61**	888.325	915.48614	0.88	0.86	0.81	0.88	1.11	963.02821	703.71	888.94705
J1-S-70X70X10	**1626.4**	1540.1	1653.1541	1.06	0.98	0.95	1.22	1.14	1709.9424	1422.36	1332.0445
J1-S-70X70X9.5	**1626.4**	1485.57	1594.616	1.09	1.02	0.99	1.30	1.19	1649.3944	1372.03	1247.644

	**Pu/PDSM**	**Pu/PDSM-RR**	**Pu/PDSM-ARR**	**Pu/PDSM-Ma**	**Pu/PDSM-Prp**
**n**	48	48	48	48	48
**mean**	0.59	0.79	0.70	0.58	1.16
**stdv**	0.13	0.01	0.02	0.25	0.05
**cov**	0.22	0.01	0.03	0.44	0.04
**Vr**	0.02	0.01	0.01	0.07	0.01
**β**	0.8	2.2	1.6	0.7	4.1
